# Epilepsy as a risk factor for hepatic encephalopathy in patients with cirrhosis: a cohort study

**DOI:** 10.1186/s12876-016-0487-3

**Published:** 2016-07-26

**Authors:** Peter Jepsen, Jakob Christensen, Karin Weissenborn, Hugh Watson, Hendrik Vilstrup

**Affiliations:** 1 Department of Hepatology and Gastroenterology, Aarhus University Hospital, Nørrebrogade 44, DK-8000 Aarhus, Denmark; 2 Department of Clinical Epidemiology, Aarhus University Hospital, Aarhus, Denmark; 3 Department of Neurology, Aarhus University Hospital, Aarhus, Denmark; 4 Department of Neurology, Hannover Medical School, Hannover, Germany; 5 Sanofi Aventis R&D, Paris, France

**Keywords:** End-stage liver disease, Neurology, Seizures, Liver failure, Prognosis

## Abstract

**Background:**

Epilepsy is associated with an increased mortality among cirrhosis patients, but the reasons are unknown. We aimed to determine whether epilepsy is a risk factor for developing hepatic encephalopathy (HE), which is a strong predictor of mortality.

**Methods:**

We used data from three randomized 1-year trials of satavaptan in cirrhosis patients with ascites. With Cox regression, we compared the hazard rates of HE grade 1–4 between those cirrhosis patients who did or did not have epilepsy. We adjusted for confounding by gender, age, cirrhosis etiology, diabetes, history of HE, Model for Endstage Liver Disease (MELD) score, serum sodium, albumin, lactulose use, rifaximin use, and benzodiazepine/barbiturate sedation. In a supplementary analysis we examined the association between epilepsy and the hazard rate of HE grade 2–4.

**Results:**

Of the 1120 cirrhosis patients with ascites, 21 (1.9 %) were diagnosed with epilepsy. These patients had better liver function at inclusion than the patients without epilepsy (median MELD score 7.9 vs. 11.4), and only one died during the trials. Nevertheless, seven patients with epilepsy had an HE episode during the follow-up, and the adjusted hazard ratio of HE grade 1–4 for patients with epilepsy vs. controls was 2.12 (95 % CI 0.99–4.55). The corresponding hazard ratio of HE grade 2–4 was 3.83 (95 % CI 1.65–8.87).

**Conclusions:**

Our findings suggest that epilepsy is associated with an increased risk of HE in patients with cirrhosis.

## Background

We have previously shown that patients with liver cirrhosis who have been given a diagnosis of epilepsy have a higher mortality than other patients with cirrhosis [1]. Unfortunately, we could not determine the reasons for that association, and no one else has studied it. However, sporadic case reports have described that hepatic encephalopathy (HE) may manifest as status epilepticus [2, 3], and since HE is a very strong predictor of mortality in cirrhosis patients [4], we thought that epilepsy might increase the risk of HE in cirrhosis patients.

Epilepsy has a prevalence of about 3 % among Danish cirrhosis patients [1], and these patients have a 1-year HE risk around 15 % [4]. Therefore it is necessary to have a large cohort of cirrhosis patients to obtain meaningful estimates of the association between epilepsy and HE risk. We had access to the complete original dataset from three large randomized controlled trials of satavaptan treatment of ascites in nearly 1200 patients with cirrhosis who were followed for 1 year, and these data presented a unique opportunity to study epilepsy as a risk factor for HE. Satavaptan had no effect on the development of HE [5].

Given this background, the aim of this study was to examine the effect of epilepsy on HE risk in patients with cirrhosis. Understanding the risk of HE in patients with epilepsy may add to our understanding of the pathogenesis of HE and improve our management of cirrhosis patients who have epilepsy.

## Methods

**Fig. 1:**
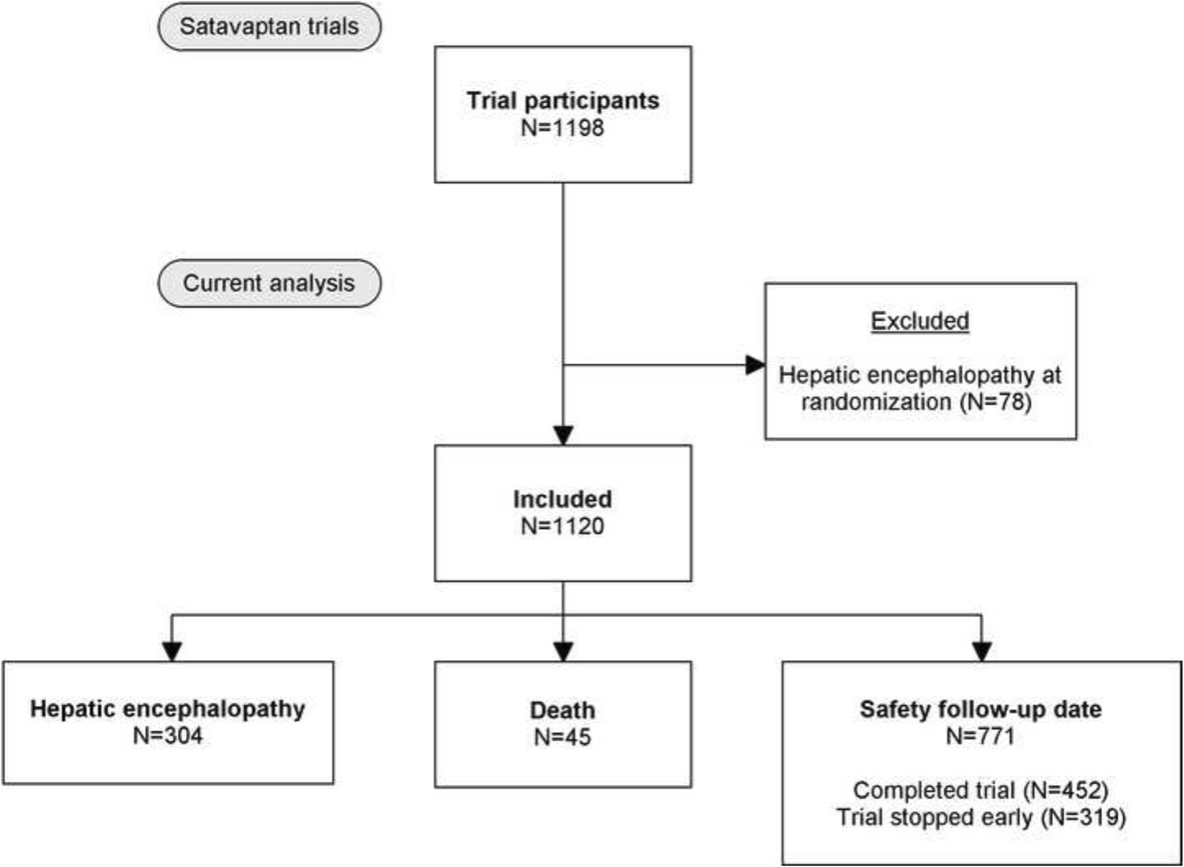
Study flow chart

Between July 2006 and December 2008 three multinational randomized controlled trials were conducted to examine whether satavaptan was efficacious in treating ascites in cirrhosis patients [6]. The three trials included patients with differing severity of ascites, but were otherwise similar and included 1,198 patients in total. Patients with a functioning transjugular intrahepatic portosystemic shunt were excluded from the trials, as were patients with variceal bleeding or spontaneous bacterial peritonitis in the 10 days before randomization. Other reasons for exclusion were: serum creatinine >151 μmol/L, serum potassium ≥5.0 mmol/L, serum sodium >143 mmol/L (because satavaptan may increase the serum sodium concentration), serum bilirubin >150 μmol/L, INR >3.0, platelets <30,000/mm^3^, neutrophils <1,000/mm^3^, hepatocellular carcinoma exceeding the Milan criteria, use of a potent modifier of the cytochrome P450 3A pathway, or use of drugs that increase the risk of Q-T interval prolongation [6]. We excluded 78 patients who were encephalopathic at the time of randomization (because they were not at risk of developing HE), leaving 1120 patients for inclusion (Fig. 1). Patients with a history of HE before randomization were included in the analyses.

### Study design

The planned treatment duration in the trials was 52 weeks, but the second and third trials were stopped early due to a poor risk-benefit ratio [6]. In all three trials, some patients discontinued the study medication prematurely, primarily due to adverse events [6]. Irrespective of the reason for discontinuation, all patients were followed for one additional week to assess drug safety. For the analysis presented here, we stopped follow-up on the date of the last drug safety assessment, i.e. 1 week after study completion or premature discontinuation.

### Data collection

Data on epilepsy, seizures, and use of antiepileptic drugs were collected at the time of randomization. The case report forms described why patients used antiepileptic drugs, but contained no details about their type of epilepsy or seizures. In our primary analysis, we categorized patients as having or not having epilepsy on the basis of epilepsy diagnoses recorded at randomization. Due to concern that epilepsy diagnoses were incorrect, we conducted a secondary analysis in which our neurologist coauthors (JC and KW) used all the available information to re-categorize patients as follows: patients with definite epilepsy (all 8 patients with a recorded diagnosis of epilepsy who were using antiepileptic drugs), patients with unspecified seizures (12 of 13 patients with a recorded diagnosis of epilepsy who were not using antiepileptic drugs; 1 of 13 patients was re-categorized as a control), patients who used antiepileptic drugs for neuropathy or other indications, and controls. We defined antiepileptic drugs by the ATC-code N03A*. Of the 12 patients with unspecified seizures, three had a history of HE before inclusion. We did not have the data to determine whether these patients’ HE episodes were related to the seizures.

During the follow-up, patients were seen every 4 weeks in their hepatology departments, and at those visits all current medications including their indications and dosages were recorded, and blood tests were taken. All HE episodes and other clinical events were recorded during the follow-up as part of the safety assessment. Every four-week visit also included a formal examination for signs and symptoms of HE by an experienced clinician, and at the same time the clinician took a history of HE episodes since the previous visit. There was no psychometric testing for minimal HE. For every HE episode clinicians recorded the severity according to the West Haven criteria [7], the dates of onset and resolution, and likely precipitants.

### Statistical analysis

For the present analysis, follow-up began at randomization and continued until the first occurrence of one of the following: onset of an HE episode (grade 1, 2, 3, or 4), death, or the safety follow-up date following study completion or premature discontinuation of the study treatment (Fig. 1). We used the chi-square test (for categorical variables) and the Mann-Whitney test (for continuous variables) to compute the p-value of the hypothesis that baseline characteristics were identical between cirrhosis patients with or without epilepsy. We used Cox proportional hazards regression to examine the association between patient category and HE hazard rate. We adjusted for confounding by patient gender, age, cirrhosis etiology (alcohol only [reference], chronic hepatitis C only, or other etiology), diabetes, history of HE before randomization, Model for Endstage Liver Disease (MELD) score, serum sodium, serum albumin, lactulose use, rifaximin use, and benzodiazepine/barbiturate sedation. This last variable was defined by the ATC codes N05BA, N05CA, N05CB, N05CD, and N05CF. Benzodiazepines and barbiturates counted as antiepileptic drugs if they were given for epilepsy (in which case they had the ATC code N03AA or N03AE), and as sedatives if they were given for anxiety or as sedation. Age, MELD score, biochemistry, and diuretics were included as continuous, linear variables, and all confounders were included as time-dependent variables. In addition, we repeated the Cox regression analysis within the stratum of patients with cirrhosis due to alcoholism.

### Sensitivity analysis

The clinical diagnosis of the low grades of HE may be uncertain so we repeated our analyses with overt HE as the outcome, i.e. HE grades 2, 3, or 4 [8]. We also repeated our analyses excluding patients who had experienced one or more HE episodes before randomization. This exclusion reduced the possibility that we included patients who had minimal HE, but it also reduced the number of patients in the study.

## Results

We included 1120 cirrhosis patients, 21 (1.9 %) of whom had a recorded diagnosis of epilepsy. During the total follow-up time of 622.2 person-years, 304 cirrhosis patients had an HE episode, and 45 died without having developed HE. The 21 cirrhosis patients who had epilepsy had less severe cirrhosis, as judged by their MELD scores (Table 1), and only one of them died during the trials. This patient died after his first HE episode, i.e. after follow-up ended in our current analysis of the trial data. Despite their favorable MELD scores, seven of the 21 cirrhosis patients with epilepsy had an HE episode during the follow-up. The HE incidence rate was 0.67 episodes per person-year for the 21 cirrhosis patients with epilepsy (seven episodes during 10.5 person-years, median 0.42 years of follow-up per patient) compared with 0.49 episodes per person-year for the 1099 cirrhosis patients without epilepsy (297 episodes during 611.7 person-years, median 0.58 years of follow-up per patient). After confounder adjustment, the rate of HE for patients with epilepsy vs. controls was more than doubled (adjusted HR = 2.12, 95 % CI 0.99–4.55), but the association was not statistically significant (Table 2). Among the 650 patients with alcoholic cirrhosis, the corresponding confounder-adjusted HR was 2.44 (95 % CI 0.96–6.17).

**Table 1:** Characteristics of the study cohort at the beginning of follow-up

	Epilepsy	Not epilepsy	*p*-value
Number of patients	21	1099	
Men (%)	18 (86 %)	760 (69 %)	0.10
Age (median, IQR)	54 (45–59)	57 (50–64)	0.04
Cirrhosis etiology			0.22
Alcohol alone (%)	16 (76 %)	634 (58 %)	
Hepatitis C alone (%)	1 (5 %)	147 (13 %)	
Other (%)	4 (19 %)	318 (29 %)	
Diabetes (%)	4 (19 %)	256 (23 %)	0.65
History of HE before randomization (%)	4 (19 %)	251 (23 %)	0.68
MELD score (median, IQR)	7.9 (4.6–11.3)	11.4 (8.1–14.4)	0.02
Sodium, mmol/L (median, IQR)	136 (133–138)	137 (134–139)	0.34
Albumin, g/L (median, IQR)	33 (29–37)	33 (29–37)	0.68
Lactulose, any dose (%)	14 (19 %)	330 (30 %)	0.28
Rifaximin, any dose (%)	0 (0 %)	27 (2 %)	0.45
Benzodiazepine/barbiturates, any dose (%)	2 (10 %)	95 (9 %)	0.89

*IQR* interquartile range, 25^th^ percentile to 75^th^ percentile

**Table 2:** Confounder-adjusted effect of a recorded diagnosis of epilepsy on the hazard rate of HE episodes grade 1, 2, 3, or 4

	Adjusted hazard ratio
Epilepsy (as recorded)	2.12 (0.99–4.55)
Male vs. female	1.07 (0.83–1.39)
Age, per 10-year increase	1.17 (1.04–1.32)
Cirrhosis etiology	
Alcohol alone (%)	1 (reference)
Hepatitis C alone (%)	1.64 (1.18–2.28)
Other (%)	1.35 (1.03–1.76)
Diabetes	1.41 (1.09–1.82)
History of HE before randomization	1.72 (1.34–2.20)
MELD score, per point	1.09 (1.07–1.11)
Sodium, per 5 mmol/L increase	0.64 (0.58–0.71)
Albumin, per 5 g/L increase	0.78 (0.70–0.87)
Lactulose, any dose vs. none	1.79 (1.41–2.28)
Rifaximin, any dose vs. none	0.59 (0.32–1.10)
Benzodiazepines/barbiturates, any dose vs. none	1.22 (0.85–1.74)

When we re-categorized patients, eight patients (0.7 %) had definite epilepsy; 12 (1.1 %) had a history of seizures but did not currently use antiepileptic drugs; 16 (1.4 %) used antiepileptic drugs for non-epilepsy indications, primarily neuropathic pain; and the remaining 1084 (96.8 %) were controls. Thus, one patient with a recorded diagnosis of epilepsy was re-categorized as a control after neurologists reviewed the available data. The eight patients with definite epilepsy used very different drug regimens: two used topiramate, one used phenytoin + gabapentin (and developed HE), one used phenytoin + valproate, one used gabapentin (and developed HE), one used carbamazepine, one used oxcarbazepine (and developed HE), and one used phenobarbital. In this analysis with four patient categories the hazard ratio estimates had wider confidence intervals because the patient groups were smaller, but the point estimate for ‘definite epilepsy’ vs. controls (adjusted HR = 2.49, 95 % CI 0.78–7.90) was essentially the same as the estimate for ‘epilepsy’ vs. controls in the primary analysis (Table 3). Also the patients we classified as having unspecified seizures and those who used antiepileptic drugs for non-epilepsy indications had higher HE rates than the controls, but their HE rates were not as high as the HE rate among patients with definite epilepsy (Table 3).

**Table 3:** Confounder-adjusted effects of definite epilepsy, unspecified seizures, and use of antiepileptic drugs for non-epilepsy indications on the hazard rate of HE episodes grade 1, 2, 3, or 4

	Adjusted hazard ratio
Patient category (after re-categorization)	
Definite epilepsy	2.49 (0.78–7.90)
Unspecified seizures	1.50 (0.47–4.76)
Antiepileptic drugs for non-epilepsy indications	1.52 (0.74–3.12)
Controls	1 (reference)
Male vs. female	1.08 (0.83–1.40)
Age, per 10-year increase	1.17 (1.03–1.32)
Cirrhosis etiology	
Alcohol alone (%)	1 (reference)
Hepatitis C alone (%)	1.65 (1.19–2.30)
Other (%)	1.35 (1.03–1.76)
Diabetes	1.38 (1.06–1.78)
History of HE before randomization	1.70 (1.33–2.18)
MELD score, per point	1.09 (1.07–1.11)
Sodium, per 5 mmol/L increase	0.63 (0.57–0.70)
Albumin, per 5 g/L increase	0.78 (0.70–0.87)
Lactulose, any dose vs. none	1.80 (1.41–2.29)
Rifaximin, any dose vs. none	0.59 (0.32–1.11)
Benzodiazepines/barbiturates, any dose vs. none	1.23 (0.86–1.76)

### Sensitivity analysis

There were 151 overt HE episodes during the follow-up, including six among the 21 cirrhosis patients with a recorded diagnosis of epilepsy. These 21 patients’ adjusted hazard ratio of overt HE vs. controls was 3.83 (95 % CI 1.65–8.87). When we re-categorized patients, the adjusted hazard ratio for patients with definite epilepsy vs. controls was 3.60 (95 % CI 0.86–15.06). It was 2.99 (95 % CI 0.92–9.75) for patients with unspecified seizures vs. controls, and 1.20 (95 % CI 0.38–3.86) for patients who used antiepileptic drugs for non-epilepsy indications vs. controls.

Finally, when we excluded the 255 patients with an HE episode before randomization the association between epilepsy and HE strengthened. The adjusted hazard ratio was 4.78 (95 % CI 1.92–11.88) for epilepsy patients vs. controls. After re-categorization it was 6.12 (95 % CI 1.47–25.44) for patients with definite epilepsy vs. controls, 2.97 (95 % CI 0.71–12.44) for patients with unspecified seizures vs. controls, and 2.76 (95 % CI 1.11–6.86) for patients who used antiepileptic drugs for non-epilepsy indications vs. controls.

## Discussion

This study was based on an unparalleled dataset with detailed clinical data on more than 1100 cirrhosis patients. We examined the association between epilepsy and HE and found similar hazard ratio estimates whether we defined epilepsy per the recorded diagnoses, or we used all available information to identify patients with definite epilepsy. Moreover, the association strengthened when we considered only episodes of overt HE, or when we excluded patients with HE episodes before inclusion. Therefore, this study provides evidence that epilepsy is a risk factor for developing HE, despite the small number of patients with epilepsy.

Our motivation for this study was to understand why epilepsy was associated with a 1.31-fold increased mortality among patients with cirrhosis [1]. We could not confirm that association in these trial data because the 21 patients with epilepsy had so well-preserved liver function that only one of them died during the 1-year follow-up in the trials. Nevertheless, epilepsy’s association with HE development in the trial data suggests that epilepsy might also have been a risk factor for mortality if patients had been followed for longer than 1 year. It is evident that this study is limited by the small number of patients with epilepsy and the short duration of follow-up, but a stronger dataset will not emerge in the foreseeable future.

The cirrhosis-related data for these analyses were rigorously defined, but the data regarding epilepsy were not. Epilepsy diagnoses were recorded by the hepatologists caring for the patients, and we had no information that explained why patients with epilepsy were not receiving antiepileptic treatment—were they uncompliant with recommended antiepileptic treatment, or did they in fact not have epilepsy? That uncertainty led us to re-categorize patients and define a patient group with definite epilepsy. It is a crucial finding that the hazard ratio estimates were similar for the larger ‘epilepsy’ and the smaller ‘definite epilepsy’ group (2.12 and 2.49, respectively). It is obvious that the confidence interval was wider for the smaller group, but the fact that the *estimates* were similar supports the conclusion that epilepsy is indeed a risk factor for developing HE.

It is likely that some of those with a recorded diagnosis of epilepsy who did not receive antiepileptic treatment (those we categorized as having ‘unspecified seizures’) did in fact have epilepsy. That possibility might explain why this group of patients had an increased HE risk. The effect of antiepileptic drugs for non-epilepsy indications was ambiguous, and we are concerned that the HE episodes might be related to the *indication* for the antiepileptic drug rather than to the drug itself. For example, some of the cirrhosis patients without epilepsy who used antiepileptic drugs suffered from diabetes or alcoholism, both of which are risk factors for neuropathy, the prevailing indication for some antiepileptic drugs. Diabetes itself is a risk factor for HE [9], and alcoholism can cause symptoms and signs that might be mistaken for HE [7]. Although we controlled for both conditions in our analysis, these patients could have *worse* diabetes or *worse* alcoholism than other patients, in which case residual confounding would cause us to overestimate the effect of antiepileptic drugs. That concern, coupled with the imprecise hazard ratio estimate, means that we do not claim that antiepileptic drugs cause HE, although the sedative properties of some antiepileptic drugs could potentially increase the risk of HE.

It is conceivable that some events perceived as HE episodes were in fact non-convulsive status epilepticus, which may resemble grade 4 HE [2, 3]. However, only one patient with epilepsy had a grade 4 HE episode, and that episode had an identified precipitant (electrolyte disturbance) and occurred in a patient who had previously had HE. We are not concerned by the risk of mistaking grade 1 HE episodes for post-ictal disorientation because our sensitivity analysis showed that epilepsy was an even stronger risk factor for *overt* HE, which is considered a more reliable clinical diagnosis [8].

Our data on the association between epilepsy and HE are merely observational and do not clarify any pathogenetic aspect of HE beyond the obvious disturbed function of the central nervous system [10]. It remains very intriguing that a condition with overshoot of excitatory neurotransmission increases the risk of developing a condition characterized by neuroinhibition [11, 12]. Our findings are probably best viewed in light of the concept of the ‘frail brain’ [13], whereby the cirrhosis patient’s brain responds with HE to *anything* that affects neurotransmission, but the links between epilepsy, antiepileptic drugs, and HE risk clearly deserve further investigation.

## Conclusions

In these data from three worldwide randomized trials in cirrhosis patients with ascites, epilepsy seemed to cause an increased risk of HE. This finding may help us understand the pathogenesis of HE and improve our clinical management of patients with cirrhosis and epilepsy.

## Abbreviations

HE, hepatic encephalopathy; MELD, model for endstage liver disease
